# Childhood adversity, social support, problematic internet use, psychological vulnerability, and pathways to non-suicidal self-injury and suicidality in adolescents and young adults: a prospective cohort study protocol

**DOI:** 10.3389/fpsyt.2026.1857155

**Published:** 2026-06-29

**Authors:** Yi-An Liao, Shih-Ying Ni, Tzu‐Jung Chiu, Chun-I Liu

**Affiliations:** Department of Psychiatry, National Taiwan University Hospital, Taipei, Taiwan

**Keywords:** adolescents and young adults, adverse childhood experience, mentalization, non-suicidal self-injury, youth mental health, suicide

## Abstract

**Background:**

Non-suicidal self-injury (NSSI) and self-harm among adolescents and young adults are increasingly recognised as major public health concerns. Although childhood adversity, family and social support, problematic internet use, psychological vulnerability, and psychiatric symptoms have each been associated with NSSI and suicidality, these factors have often been examined separately. Less is known about how environmental adversity, interpersonal support, and psychological vulnerability interact within an integrated developmental pathway leading to NSSI and suicidality.

**Methods and analysis:**

Adolescents and young adults aged 13–29 years with a recent history of self-harm or suicidality will be recruited from National Taiwan University Hospital. Community participants without an established psychiatric diagnosis will be recruited from collaborating schools as controls. Participants will complete self-report and caregiver-report measures assessing NSSI, suicidality, childhood adversity, family and social support, problematic internet use, reflective functioning, self-efficacy, impulsivity, and internalising and externalising symptoms. Descriptive and group-comparison analyses will compare psychosocial and clinical characteristics between the clinical and community groups. The primary analysis will use structural equation modelling within the NSSI group to examine whether childhood adverse environment, inadequate family and social support, and problematic internet use are associated with NSSI and suicidality through psychological vulnerability and downstream internalising and externalising problems. Sample size estimation was based on Monte Carlo simulation-based power analysis in R. Exploratory network analysis will identify observed variables most directly connected to NSSI and suicidality and detect bridge nodes across psychosocial and symptom domains.

**Discussion:**

Previous studies have identified multiple psychosocial and psychological correlates of NSSI and suicidality, but these factors have rarely been examined within an integrated developmental framework. By jointly examining early adversity, interpersonal support, problematic internet use, mentalization-related vulnerability, impulsivity, self-efficacy, and psychiatric symptoms, this study may clarify how distal environmental risks and proximal psychological vulnerabilities become clinically connected in young people with self-harm. Findings may inform earlier, mechanism-informed, and context-sensitive prevention strategies targeting upstream vulnerabilities before progression to repeated NSSI or suicidal behaviour.

## Background

Non-suicidal self-injury (NSSI) refers to the deliberate destruction or alteration of one’s own body tissue without suicidal intent and is increasingly recognised as a major mental health concern in adolescents and young adults (1, 2). NSSI is common in youth populations, and recent meta-analytic evidence suggests that its prevalence, repetition, and clinical severity may be increasing, with important implications for later suicidality and broader psychiatric burden (3, 4).

A broad range of factors have been associated with NSSI, including psychiatric symptoms, childhood adversity, interpersonal difficulties, impulsivity, and problematic internet-related behaviours (5). However, many previous studies have examined these correlates in isolation, whereas NSSI in adolescents and young adults is more likely to arise from the interaction between intrapersonal vulnerability and adverse environmental contexts (6–9).

At the individual level, theory of mind and related mentalization processes may provide a useful framework for understanding vulnerability to NSSI (10, 11). Deficits in reflective functioning and other self-regulatory processes, including impulsivity and reduced coping-related self-efficacy, have been linked to emotional and behavioural dysregulation and may increase susceptibility to self-injurious behaviour (12–16).

At the environmental level, childhood maltreatment and other adverse childhood experiences are well-established risk factors for self-harm and may shape long-term vulnerability through their effects on development, cognition, and emotion regulation (17–20). Family and social support may serve as protective developmental scaffolds, whereas poor family functioning and inadequate perceived support have been associated with greater NSSI risk (21–24). In addition, problematic internet use, cyberbullying, and exposure to self-injury-related online content have emerged as relevant contemporary correlates of NSSI in young people (6, 25–27). From a biopsychosocial developmental perspective, childhood adversity may be conceptualised not only as a distal stressor, but also as a developmental condition that affects emotion regulation, attachment security, reflective functioning, and access to protective relationships (18, 28). Inadequate family and social support may therefore serve as mechanisms through which adversity becomes expressed as psychological vulnerability, while impaired mentalization may limit adolescents’ capacity to understand and verbalise distress (29).

In Taiwan, NSSI and suicidality among adolescents and young adults have been recognised as an emerging public health concern (30). National statistics indicate a substantial upward trend in both suicide deaths and suicide-related reports among Taiwanese youth aged 15–24 years between 2015 and 2024 (31). However, most existing studies have focused on prevalence or isolated risk factors rather than integrated developmental pathways. To address this public health concern in Taiwan, an integrated and comprehensive approach is warranted to examine developmental pathways in Taiwanese youth and to clarify the links between psychosocial adversity, psychological vulnerability, NSSI, and suicidality.

The present study is informed by a biopsychosocial developmental framework. In this framework, self-harm and suicidality are conceptualised not simply as behavioural responses to stress exposure, but as outcomes shaped by the interplay between pre-existing biological vulnerability, psychological capacities, and social and environmental contexts (32–35). Childhood adversity in the context of inadequate family and social support may contribute to psychological vulnerabilities involving emotion regulation, attachment, mentalization, and bodily self-experience (29), which may in turn manifest through internalising and externalising problems and be associated with NSSI and suicidality (28, 36). Although NSSI and suicidality are distinct constructs, they are clinically interconnected (37). Both may share developmental and psychological risk factors, and longitudinal evidence suggests that NSSI is associated with increased later vulnerability to suicidal thoughts and behaviours (38). Therefore, this study assesses NSSI and suicidality separately using the ISAS and C-SSRS, while examining them as related outcomes within the same developmental framework.

**Figure 1:**
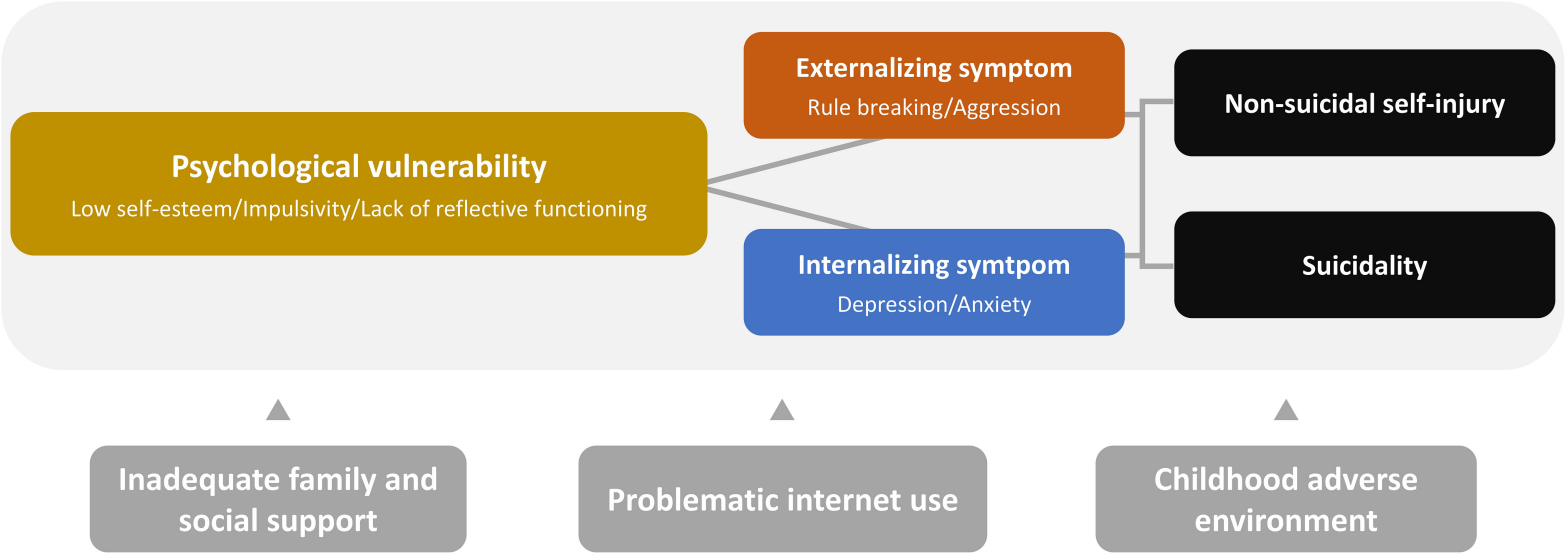
Study framework. Inadequate family and social support, problematic internet use, and childhood adverse environment display as environmental factors, which has interplay with all psychological diathesis, internalising and externalising symptoms, non-suicidal self-injury and suicide.

Based on this framework, we propose that childhood adverse environment, inadequate family and social support, and problematic internet use may contribute to psychological vulnerability, which may in turn manifest through internalising and externalising problems and be associated with NSSI and suicidality (**Figure 1**). Accordingly, this protocol aims to examine these domains within a single integrative model in Taiwanese adolescents and young adults.

## Methods

### Reporting guideline

This study protocol was prepared in accordance with the Strengthening the Reporting of Observational Studies in Epidemiology (STROBE) checklist where applicable (39). The checklist was used to guide the reporting of the study design, setting, participants, eligibility criteria, variables, data sources and measurements, sample size considerations, planned statistical methods, ethical procedures, and anticipated interpretation of findings.

### Aims

The aims of this study are threefold. First, we aim to compare proposed psychosocial risk factors between adolescents and young adults with NSSI and community controls in Taiwan, including childhood adversity, family and social support, problematic internet use, and psychological vulnerability. Second, we aim to test a hypothesised pathway model within the NSSI group using structural equation modelling (SEM), specifically examining whether childhood adverse environment, family and social support, and problematic internet use are associated with NSSI and suicidality through psychological vulnerability, internalising problems, and externalising problems. Third, we aim to use exploratory network analysis to identify observed psychosocial and symptom variables that are most directly connected to NSSI and suicidality, and to detect potential bridge nodes linking distal adversity, interpersonal support, problematic internet use, psychological vulnerability, and symptom domains.

### Participants and procedures

We will recruit adolescents and young adults aged 13–29 years with a history of self-harm or suicidality within the past year from the Department of Psychiatry at National Taiwan University Hospital, Taipei, Taiwan. Recruitment and baseline data collection are planned to take place from July 2025 to June 2027. Community participants without an established psychiatric diagnosis will be recruited from collaborating schools in Taipei as controls. Individuals with schizophrenia-spectrum disorders, bipolar I disorder, substance use disorder, or physical illness with limited intellectual ability that precludes completion of interviews or questionnaires will be excluded.

All participants will receive a full explanation of the study procedures before providing informed consent. For minors, consent will be obtained from a parent or legal guardian, and assent will be obtained from the participant. All the participants will complete the study questionnaires either on paper or online.

Because the clinical group includes adolescents and young adults with recent self-harm or suicidality, participants in the clinical group will be assessed in a clinical setting by trained psychiatrists. If current suicidal intent, recent suicidal behaviour, escalating self-harm, or severe distress is identified, the research assessment will be discontinued immediately, and the participant will be referred for clinical evaluation and appropriate intervention, including safety planning, caregiver involvement, urgent referral, or emergency management.

For community controls, recruitment will be conducted through collaborating high schools and colleges. Participants who report clinically significant suicidal ideation or self-harm will be referred to school counselling or mental health services, with psychiatric consultation when urgent risk is suspected. Participants may pause or withdraw at any time, and all data will be de-identified and accessible only to authorised study personnel. The study protocol was approved by the Research Ethics Committee of National Taiwan University Hospital (REC No. 202505059RINC).

Because the primary objective of this study is to test the hypothesised SEM within the NSSI group, sample size estimation was based on the SEM rather than on between-group comparisons. A Monte Carlo simulation-based power analysis was conducted in R using the lavaan package. The simulation evaluated power for key direct and indirect effects in the hypothesised model and the proportion of replications achieving acceptable model fit. Based on these analyses, a minimum of 120 participants with NSSI was considered sufficient. To account for potentially unusable responses and deviations from the assumed model conditions, the planned recruitment target for the NSSI group was increased to 150 participants. Community controls will be used primarily for descriptive and group-comparison analyses.

### Measures

The study includes both self-report and caregiver-report measures. Suicidality will be assessed using the Columbia-Suicide Severity Rating Scale (C-SSRS), and NSSI will be assessed using the Inventory of Statements About Self-Injury (ISAS). Problematic internet use will be assessed using the Smartphone Addiction Inventory Short Form (SPAI-SF) and the Bergen Social Media Addiction Scale (BSMAS). Psychological vulnerability will be assessed using the Reflective Functioning Questionnaire (RFQ), the General Self-Efficacy Scale (GSES), and the Barratt Impulsiveness Scale (BIS). Family and social support will be assessed using the Inventory of Parent and Peer Attachment (IPPA) and the Multidimensional Scale of Perceived Social Support (MSPSS). Childhood adversity will be assessed using the Childhood Trauma Questionnaire–Short Form (CTQ-SF). Internalising and externalising problems will be assessed using the Beck Anxiety Inventory (BAI), Beck Depression Inventory (BDI), Brief Symptom Rating Scale (BSRS-5), and the Child Behavior Checklist (CBCL), Youth Self Report (YSR), or Young Adult Behavior Checklist (YABCL), as appropriate. Among these measures, the CBCL and YABCL will be completed by caregivers, whereas the remaining questionnaires will be self-administered. Validated Chinese versions will be used for all instruments. A detailed summary of the study instruments, including their analytic domains, informants, number of items, score ranges, coding directions, analytic roles, and Chinese validation references, is provided in **Supplementary Table 1**.

### Statistical analysis

Descriptive statistics will be used to summarise demographic characteristics and psychometric measures. Continuous variables will be presented as means and standard deviations, and categorical variables as frequencies and percentages. Group differences between participants with NSSI and community controls will be examined using independent t tests or chi-squared tests, as appropriate. Effect sizes will be reported using Cohen’s d for continuous variables and Cohen’s ω for categorical variables. Pearson’s correlation analysis will be used to examine bivariate relationships among key variables. A two-sided p value of less than 0.05 will be considered statistically significant.

SEM will be used to examine the hypothesised relationships among childhood adverse environment, family and social support, problematic internet use, psychological vulnerability, internalising symptoms, externalising problems, NSSI, and suicidality within the NSSI group. Analyses will be conducted in two stages: evaluation of the measurement model followed by testing of the structural model. All scale scores will be coded such that higher scores represent greater adversity, poorer support, greater vulnerability, or more severe psychopathology. Accordingly, GSES scores will be reverse-coded so that lower self-efficacy reflects greater psychological vulnerability. MSPSS and IPPA scores will also be reverse-coded when modelling inadequate family and social support.

In the measurement model, psychological vulnerability will be specified as a latent construct indicated by the RFQ, reverse-coded GSES, and BIS. Internalising symptoms will be specified as a latent construct indicated by the CBCL internalising score, BSRS-5, BAI, and BDI. Inadequate family and social support will be modelled as a latent construct indicated by the reverse-coded MSPSS, IPPA-peer, and IPPA-parent scores. Problematic internet use will be modelled as a latent construct indicated by the SPAI-SF and BSMAS. Childhood adverse environment will be represented by the CTQ-SF total score. Externalising problems will be represented by the CBCL externalising score. NSSI will be assessed using the ISAS, and suicidality will be assessed using the C-SSRS.

In the structural model, childhood adverse environment and problematic internet use will be allowed to covary. Both variables will be specified as predictors of inadequate family and social support and psychological vulnerability. Inadequate family and social support will in turn be specified as a predictor of psychological vulnerability. Psychological vulnerability will be modelled as a predictor of both internalising and externalising problems. Internalising and externalising problems will be allowed to covary and will each be specified as predictors of NSSI and suicidality. NSSI and suicidality will also be allowed to covary. Direct, indirect, and total effects will be estimated, and hypothesised mediation pathways will be evaluated using bias-corrected bootstrap confidence intervals based on 5, 000 resamples.

**Figure 2:**
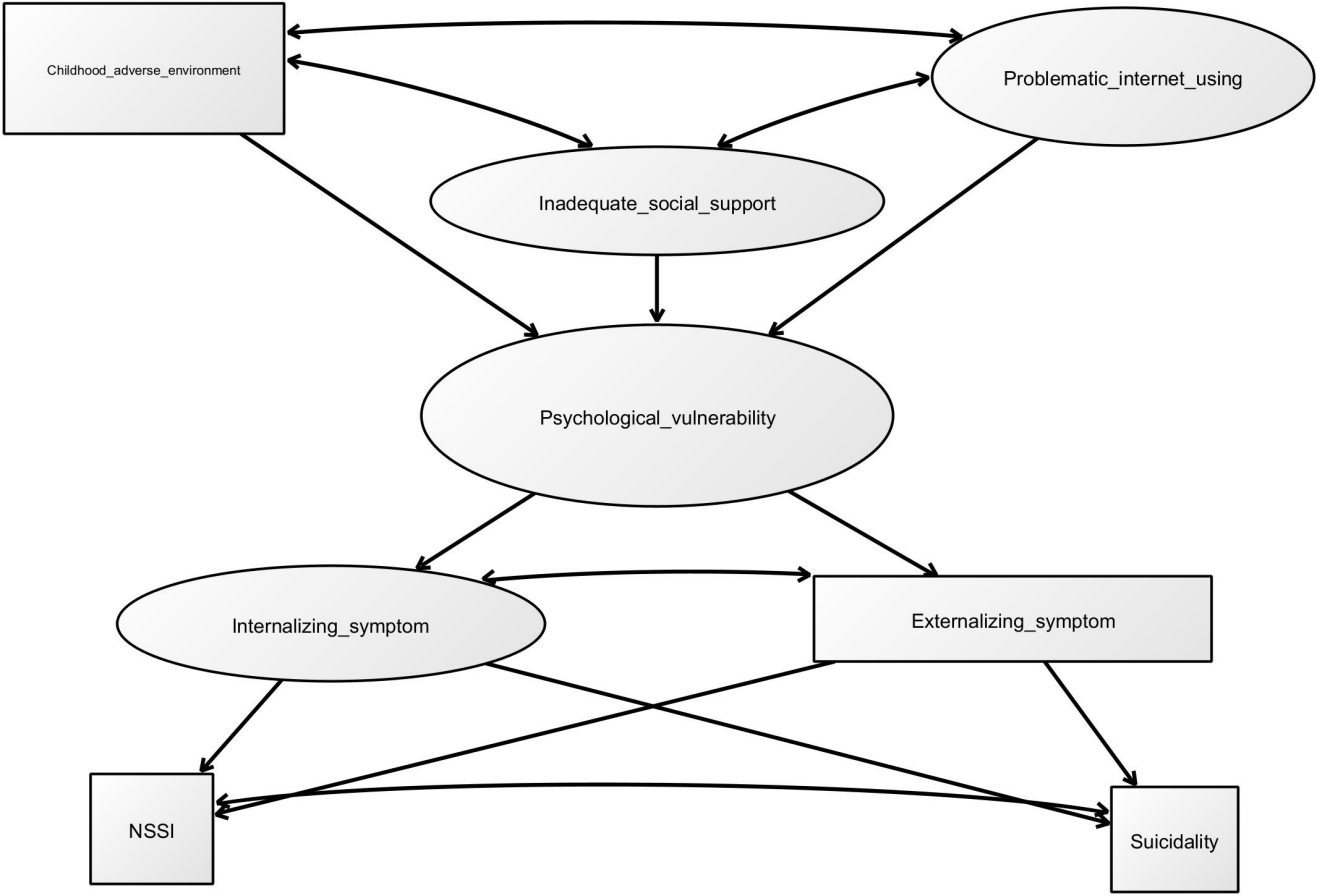
Path diagram of the proposed structured equation model.

Model parameters will be estimated using robust maximum likelihood estimation. Model fit will be evaluated using the comparative fit index (CFI), Tucker-Lewis index (TLI), root mean square error of approximation (RMSEA), and standardised root mean square residual (SRMR). Acceptable model fit will be defined as CFI and TLI >0.90 and RMSEA and SRMR <0.08, with more stringent criteria of CFI and TLI >0.95 and RMSEA <0.06 indicating good fit. Standardised path coefficients, 95% confidence intervals, and two-sided p values will be reported. All analyses will be conducted in RStudio using the lavaan package. The path diagram of the proposed model was shown in **Figure 2**.

To complement the SEM, exploratory network analysis will be conducted within the NSSI group to examine direct associations among observed psychosocial and symptom variables. Variables will be grouped into predefined communities reflecting childhood adversity, family and social support, problematic internet use, psychological vulnerability, internalising symptoms, externalising problems, NSSI, and suicidality. A Gaussian graphical model will be estimated using the qgraph package in R, and 1-step bridge expected influence will be used to identify bridge nodes across communities. Network stability will be evaluated using bootstrapping procedures.

## Discussion

The present protocol is designed to address an important gap in the NSSI literature by integrating multiple domains that are often examined separately, including childhood adversity, family and social support, problematic internet use, psychological vulnerability, and internalising and externalising problems, within a single conceptual framework. Rather than focusing only on isolated correlates of self-harm, this study aims to evaluate how these factors may operate together in adolescents and young adults with history of self-harm or suicide, while also providing contextual comparison with community controls. Although the present study does not directly assess perinatal or infancy-related factors, its focus on psychological development is consistent with birth-centred developmental perspectives, which suggest that disruptions in early relational environments may later emerge as difficulties in emotional containment, self-representation, and maladaptive bodily regulation during adolescence (40, 41).

If supported by the empirical findings, the proposed framework may clarify not only which factors are associated with NSSI and suicidality, but also how these factors become clinically connected during development. From this perspective, self-harm and suicidality can be understood as outcomes of developmental interactions among early adversity, insufficient family and social support, and psychological vulnerability. When distress cannot be adequately understood, verbalised, or regulated within supportive relationships, NSSI may function as a maladaptive attempt to regulate affect, restore control, or communicate psychological pain. Clinically, this framework suggests that prevention should address not only self-harm behaviours and psychiatric symptoms, but also upstream relational and developmental vulnerabilities, including family communication, social support, reflective functioning, and isolation. In this way, the protocol is intended to move beyond a single-risk-factor approach and to test a more developmentally informed model of vulnerability to both NSSI and suicidality.

Several practical and operational issues are particularly relevant to the conduct of this study. First, the target population includes adolescents and young adults with a recent history of self-harm or suicide, which requires a clinically sensitive recruitment process and careful ethical oversight. The study therefore embeds recruitment within a psychiatric setting for the case group, with diagnostic assessment conducted by a trained psychiatrist before questionnaire administration. Second, the control group is recruited through collaborating schools, which improves feasibility and access to community participants, but also means that the clinical and control samples arise from different recruitment contexts. This design reflects a pragmatic balance between clinical relevance and recruitment feasibility. In addition, the use of both self-report and caregiver-completed measures may also enhance the ecological validity of the study by capturing complementary perspectives on emotional and behavioural problems.

This protocol also has several methodological strengths. The proposed SEM is explicitly theory driven and distinguishes measurement from structural components, allowing the study to examine whether childhood adverse environment, inadequate support, and problematic internet use are associated with NSSI and suicidality through psychological vulnerability and symptom dimensions. In parallel, the planned network analysis is not intended to duplicate the SEM, but to complement it by identifying direct associations among observed psychosocial and symptom variables and by detecting potential bridge nodes linking adversity, support, vulnerability, and outcomes. This combination may provide a richer account of NSSI-related processes than either method alone. Moreover, the primary SEM sample size was informed by Monte Carlo simulation rather than by a simple rule-of-thumb approach, with the final recruitment target increased to 150 participants with NSSI to preserve analytic adequacy under realistic study conditions.

Several limitations should be considered. The NSSI group will be recruited from a single tertiary psychiatric centre, and the findings may therefore be more representative of help-seeking or clinically referred young people than of individuals in the broader community. In addition, although the inclusion of participants aged 13 to 29 years allows the study to examine a broader youth developmental spectrum, this age range also introduces heterogeneity in developmental stage, family dependence, peer dynamics, and digital behaviour. The study also relies heavily on self-report questionnaire-based assessments, which may be influenced by reporting bias. Finally, although healthy controls are included for descriptive and group-comparison purposes, the protocol is primarily powered for the SEM within the NSSI group; accordingly, comparisons involving controls should be interpreted as complementary rather than as the principal test of the hypothesised structural model.

By empirically testing this framework, the present study may help move clinical practice beyond the management of self-harm episodes alone and towards the identification of upstream factors that give rise to psychological vulnerability in adolescents and young adults. Specifically, by clarifying how dysfunctional parenting, inadequate social support, problematic internet use, and childhood adverse environments contribute to vulnerability traits such as low self-efficacy, impulsivity, and poor reflective functioning, this framework may help clinicians detect risk at an earlier and more developmentally meaningful stage. Such an approach has important preventive implications, as interventions targeting these underlying ecological and developmental determinants may reduce the likelihood that psychological vulnerability later manifests as internalising or externalising problems, and subsequently progresses to NSSI or suicidal behaviour. If the proposed framework is supported, it may inform earlier, mechanism-informed, and context-sensitive prevention strategies aimed not only at treating NSSI, but also at interrupting the developmental pathways that lead to self-harm and suicide.
