# Supplementary material for: Childhood adversity, social support, problematic internet use, psychological vulnerability, and pathways to non-suicidal self-injury and suicidality in adolescents and young adults: a prospective cohort study protocol
Source: Front Psychiatry. 2026 Jun 29;17:1857155. doi: 10.3389/fpsyt.2026.1857155 (PMC13360428; doi:10.3389/fpsyt.2026.1857155)
Supplement: Supplementary file 1 [file Table1.docx]

Supplementary Table 1. Study instruments and their mapping to the analytic model

| **Analytic domain** | **Instrument / indicator** | **Informant** | **No. of items** | **Score range / response format** | **Variable type in analysis** | **Coding direction** | **Primary analytic role** | **Chinese validation / reference** |
| --- | --- | --- | --- | --- | --- | --- | --- | --- |
| Suicidality | C-SSRS | Self-report | 6 | Dichotomous Yes/No items | Observed variable | Higher = greater suicidality | Outcome; network node | (Ji et al., 2023) |
| NSSI | ISAS | Self-report | 12 behavior items + 39 function items | Behavior frequency; function items rated 0–2 | Observed variable | Higher = more frequent / more severe NSSI | Outcome; network node | (Tian et al., 2025) |
| Problematic internet use | SPAI-SF | Self-report | 10 | 4-point Likert (1–4) | Latent indicator | Higher = more problematic smartphone use | Exogenous predictor; network node | (Lin et al., 2017) |
| Problematic internet use | BSMAS | Self-report | 6 | 5-point Likert (1–5) | Latent indicator | Higher = more problematic social media use | Exogenous predictor; network node | (Leung et al., 2020) |
| Psychological vulnerability | BIS | Self-report | 8 | 4-point scale (1–4) | Latent indicator | Higher = greater impulsivity / vulnerability | Mediator; network node | (Yao et al., 2007) |
| Psychological vulnerability | RFQ | Self-report | 8 | 7-point Likert (1–7) | Latent indicator | Coded such that higher = greater vulnerability | Mediator; network node | (Chen et al., 2024) |
| Psychological vulnerability | GSES | Self-report | 10 | 4-point Likert (1–4) | Latent indicator | **Reverse-coded**; higher = lower self-efficacy / greater vulnerability | Mediator; network node | (Zhang & Schwarzer, 1995) |
| Inadequate social support | MSPSS | Self-report | 12 | 7-point Likert (1–7) | Latent indicator | **Reverse-coded**; higher = poorer support | Exogenous predictor / mediator; network node | (Chou, 2000) |
| Inadequate social support | IPPA-parent | Self-report | 50 Subscale from 75-item IPPA | 5-point Likert (1–5) | Latent indicator | **Reverse-coded**; higher = poorer support | Exogenous predictor / mediator; network node | (Zhang et al., 2011) |
| Inadequate social support | IPPA-peer | Self-report | 25 Subscale from 75-item IPPA | 5-point Likert (1–5) | Latent indicator | **Reverse-coded**; higher = poorer support | Exogenous predictor / mediator; network node | (Zhang et al., 2011) |
| Childhood adverse environment | CTQ-SF total score | Self-report | 28 | 5-point Likert (1–5) | Observed variable | Higher = more severe childhood adversity | Exogenous predictor; network node | (Cheng et al., 2018) |
| Internalizing symptoms | BAI | Self-report | 21 | 4-point scale (0–3) | Latent indicator | Higher = more severe anxiety symptoms | Mediator; network node | (Che et al., 2006) |
| Internalizing symptoms | BDI | Self-report | 21 | Four statements scored 0–3 | Latent indicator | Higher = more severe depressive symptoms | Mediator; network node | (Yeung et al., 2002) |
| Internalizing symptoms | BSRS-5 | Self-report | 5 | 5-point scale (0–4) | Latent indicator | Higher = more severe psychological symptoms | Mediator; network node | (Lee et al., 2003) |
| Internalizing symptoms | CBCL / YSR / YABCL internalizing score | CBCL/YABCL: caregiver-report; YSR: self-report | 113 | 3-point scale (0–2) | In SEM, **CBCL-internalising** is a latent indicator | Higher = more severe internalizing problems | Mediator; network node | (Ivanova et al., 2007) |
| Externalizing symptoms | CBCL / YSR / YABCL externalizing score | CBCL/YABCL: caregiver-report; YSR: self-report | 113 | 3-point scale (0–2) | In SEM, **CBCL-externalising** is an observed variable | Higher = more severe externalizing problems | Mediator; network node | (Ivanova et al., 2007) |

**Reference**

Che, H.-H., Lu, M.-L., Chen, H.-C., Chang, S.-W., & Lee, Y.-J. (2006). Validation of the Chinese Version of the Beck Anxiety Inventory. *Formosan Journal of Medicine*, *10*(4), 447–454. <https://doi.org/10.6320/FJM.2006.10(4).05>

Chen, W., Li, Y., Jahrami, H., Wang, W., Zheng, W., Huang, Y., Bragazzi, N. L., & Gao, W. (2024). Validation of the Three-Factor Reflective Functioning Questionnaire for Youth in a Chinese Adolescent Sample. *Neuropsychiatr Dis Treat*, *20*, 1799–1808. <https://doi.org/10.2147/ndt.S475659>

Cheng, Y.-C., Chen, C.-H., Chou, K.-R., Kuo, P.-H., & Huang, M.-C. (2018). Reliability and Factor Structure of the Chinese Version of Childhood Trauma Questionnaire-Short Form in Patients with Substance Use Disorder. *Taiwanese Journal of Psychiatry*, *32*(1), 52–62.

Chou, K.-L. (2000). Assessing Chinese adolescents’ social support: the multidimensional scale of perceived social support. *Personality and Individual Differences*, *28*(2), 299–307. <https://doi.org/https://doi.org/10.1016/S0191-8869(99)00098-7>

Ivanova, M. Y., Dobrean, A., Dopfner, M., Erol, N., Fombonne, E., Fonseca, A. C., Frigerio, A., Grietens, H., Hannesdottir, H., Kanbayashi, Y., Lambert, M., Achenbach, T. M., Larsson, B., Leung, P., Liu, X., Minaei, A., Mulatu, M. S., Novik, T. S., Oh, K. J.,…Chen, W. J. (2007). Testing the 8-syndrome structure of the child behavior checklist in 30 societies. *J Clin Child Adolesc Psychol*, *36*(3), 405–417. <https://doi.org/10.1080/15374410701444363>

Ji, Y., Liu, X., Zheng, S., Zhong, Q., Zheng, R., Huang, J., & Yin, H. (2023). Validation and application of the Chinese version of the Columbia-Suicide Severity Rating Scale: Suicidality and cognitive deficits in patients with major depressive disorder. *J Affect Disord*, *342*, 139–147. <https://doi.org/10.1016/j.jad.2023.09.014>

Lee, M. B., Liao, S. C., Lee, Y. J., Wu, C. H., Tseng, M. C., Gau, S. F., & Rau, C. L. (2003). Development and verification of validity and reliability of a short screening instrument to identify psychiatric morbidity. *J Formos Med Assoc*, *102*(10), 687–694.

Leung, H., Pakpour, A. H., Strong, C., Lin, Y. C., Tsai, M. C., Griffiths, M. D., Lin, C. Y., & Chen, I. H. (2020). Measurement invariance across young adults from Hong Kong and Taiwan among three internet-related addiction scales: Bergen Social Media Addiction Scale (BSMAS), Smartphone Application-Based Addiction Scale (SABAS), and Internet Gaming Disorder Scale-Short Form (IGDS-SF9) (Study Part A). *Addict Behav*, *101*, 105969. <https://doi.org/10.1016/j.addbeh.2019.04.027>

Lin, Y. H., Pan, Y. C., Lin, S. H., & Chen, S. H. (2017). Development of short-form and screening cutoff point of the Smartphone Addiction Inventory (SPAI-SF). *Int J Methods Psychiatr Res*, *26*(2). <https://doi.org/10.1002/mpr.1525>

Tian, X., Huang, H., Lu, W., Zhang, R., Wang, R., Li, X., Li, D., Gao, Y., Wu, S., Xu, G., Shao, R., & Lin, K. (2025). Non-suicidal self-injury in adolescence: a validation of the Chinese version of the Inventory of Statements About Self-Injury in student populations. *Front Psychiatry*, *16*, 1510681. <https://doi.org/10.3389/fpsyt.2025.1510681>

Yao, S., Yang, H., Zhu, X., Auerbach, R. P., Abela, J. R., Pulleyblank, R. W., & Tong, X. (2007). An examination of the psychometric properties of the Chinese version of the Barratt Impulsiveness Scale, 11th version in a sample of Chinese adolescents. *Percept Mot Skills*, *104*(3 Pt 2), 1169–1182. <https://doi.org/10.2466/pms.104.4.1169-1182>

Yeung, A., Howarth, S., Chan, R., Sonawalla, S., Nierenberg, A. A., & Fava, M. (2002). Use of the Chinese version of the Beck Depression Inventory for screening depression in primary care. *J Nerv Ment Dis*, *190*(2), 94–99. <https://doi.org/10.1097/00005053-200202000-00005>

Zhang, J. X., & Schwarzer, R. (1995). Measuring optimistic self-beliefs: A Chinese adaptation of the General Self-Efficacy Scale. *Psychologia: An International Journal of Psychology in the Orient*, *38*(3), 174–181.

Zhang, Y.-L., Zhang, Y.-L., Zhang, Y.-X., Wang, J.-L., & Hung, C.-Y. (2011). Reliability and validity of Chinese version of Revised Inventory of Parent and Peer Attachment in junior students. *Chinese Mental Health Journal*, *25*(1), 66–70.
