# Supplementary material for: Childhood adversity, social support, problematic internet use, psychological vulnerability, and pathways to non-suicidal self-injury and suicidality in adolescents and young adults: a prospective cohort study protocol
Source: Front Psychiatry. 2026 Jun 29;17:1857155. doi: 10.3389/fpsyt.2026.1857155 (PMC13360428; doi:10.3389/fpsyt.2026.1857155)
Supplement: Supplementary file 2 [file Table2.docx]

**STROBE Checklist**

Manuscript: Childhood adversity, social support, problematic internet use, psychological vulnerability, and pathways to non-suicidal self-injury and suicidality in adolescents and young adults: study protocol

**Note:** Because this is a protocol rather than a completed empirical report, results-related items are marked as not applicable at the protocol stage and should be completed in the final study report.

| **Section** | **Item** | **STROBE recommendation** | **Manuscript location** | **Assessment / comments** |
| --- | --- | --- | --- | --- |
| Title and abstract | 1a | Indicate the study’s design with a commonly used term in the title or the abstract | Title; Abstract—Methods | Reported. The title currently leaves the study-design term and the abstract describes enrolment-time questionnaire and interview assessment. |
| Title and abstract | 1b | Provide in the abstract an informative and balanced summary of what was done and what was found | Abstract—Background, Methods, Discussion | Protocol-stage adaptation. The abstract summarises the rationale, participants, baseline measurements, planned analyses, expected findings, and implications. Because this is a protocol, empirical findings are not yet reported; expected results/implications are described instead. |
| Introduction | 2 | Explain the scientific background and rationale for the investigation being reported | Background | Reported. The manuscript explains the importance of NSSI and suicidality in youth, limitations of single-risk-factor studies, and the rationale for integrating childhood adversity, interpersonal support, problematic internet use, psychological vulnerability, and symptoms. |
| Introduction | 3 | State specific objectives, including any prespecified hypotheses | Background final paragraph; Methods—Aims | Reported. The manuscript states three aims: clinical-control comparisons, SEM testing of hypothesised pathways within the NSSI group, and exploratory network analysis to identify variables and bridge nodes linked to NSSI and suicidality. |
| Methods | 4 | Present key elements of study design early in the paper | Abstract—Methods; Methods—Reporting guideline; Methods—Aims; Participants and procedures | Reported. The manuscript presents a protocol for baseline data collection in a clinical group and community controls, using questionnaires and diagnostic interview at enrolment, followed by planned SEM and network analysis. |
| Methods | 5 | Describe the setting, locations, and relevant dates, including periods of recruitment, exposure, follow-up, and data collection | Participants and procedures | Reported. Setting and locations are described: Department of Psychiatry, National Taiwan University Hospital, Taipei, Taiwan, and collaborating schools in Taipei. Recruitment and baseline data collection are planned from July 2025 to June 2027. |
| Methods | 6a | Give the eligibility criteria, and the sources and methods of selection of participants | Participants and procedures | Reported. The manuscript defines the clinical sample, age range, recent self-harm/suicidality criterion, hospital recruitment source, community control recruitment source, and exclusion criteria. |
| Methods | 7 | Clearly define all outcomes, exposures, predictors, potential confounders, and effect modifiers. Give diagnostic criteria, if applicable | Measures; Statistical analysis | Reported for major outcomes and predictors. Outcomes include NSSI and suicidality; exposures/predictors include childhood adversity, family/social support, problematic internet use, psychological vulnerability, and internalising/externalising problems. Effect modifiers are not currently planned. |
| Methods | 8 | For each variable of interest, give sources of data and details of methods of assessment (measurement). Describe comparability of assessment methods if there is more than one group | Measures; Supplementary Table 1 | Reported. The manuscript lists self-report, caregiver-report, and interview instruments for NSSI, suicidality, problematic internet use, psychological vulnerability, family/social support, childhood adversity, and symptoms. It states that validated Chinese versions will be used and that instrument details are in Supplementary Table 1. Consider adding that identical participant-administered questionnaires will be used across groups where applicable. |
| Methods | 9 | Describe any efforts to address potential sources of bias | Participants and procedures; Measures; Statistical analysis; Discussion—limitations | Reported. The manuscript addresses clinical safety procedures, trained psychiatrist assessment in the clinical group, validated Chinese instruments, de-identification, and discussion of recruitment-context and self-report bias. |
| Methods | 10 | Explain how the study size was arrived at | Participants and procedures—sample size paragraph; Abstract—Methods | Reported. Sample size estimation is based on Monte Carlo simulation power analysis for the primary SEM within the NSSI group, with a minimum of 120 and a planned target of 150 participants to account for unusable responses and deviations from assumptions. Community controls are used primarily for descriptive and group-comparison analyses. |
| Methods | 11 | Explain how quantitative variables were handled in the analyses. If applicable, describe which groupings were chosen and why | Statistical analysis | Reported. The manuscript describes continuous and categorical summaries, group comparisons, effect sizes, correlation analyses, scale coding, reverse coding, latent constructs, observed variables, robust maximum likelihood estimation, and fit criteria. Categorisation of continuous variables is not planned unless later specified. |
| Methods | 12a | Describe all statistical methods, including those used to control for confounding | Statistical analysis | Reported. The manuscript specifies descriptive statistics, independent t tests, chi-squared tests, effect sizes, Pearson correlations, SEM, bootstrapped indirect effects, model-fit criteria, and exploratory network analysis. |
| Methods | 12b | Describe any methods used to examine subgroups and interactions | Statistical analysis | Not planned. The manuscript does not prespecify subgroup or interaction analyses. |
| Methods | 12c | Explain how missing data were addressed | Statistical analysis | Not applicable. For the study applied a cross-sectional design, only participants with complete and analysable baseline data for the relevant variables will be included in each analysis. |
| Methods | 12d | If applicable, describe analytical methods taking account of sampling strategy | Participants and procedures; Statistical analysis | Reported. Participants are recruited from one tertiary psychiatric centre and collaborating schools rather than by probability sampling. |
| Methods | 12e | Describe any sensitivity analyses | Statistical analysis | Not planned. No sensitivity analyses are prespecified in the present protocol. |
| Results | 13a | Report numbers of individuals at each stage—eg numbers potentially eligible, examined for eligibility, confirmed eligible, included in the study, and analysed | Not applicable at protocol stage | Not applicable at protocol stage. The completed study should report numbers screened, eligible, consented/assented, enrolled, excluded, and analysed in each group. |
| Results | 13b | Give reasons for non-participation at each stage | Not applicable at protocol stage | Not applicable at protocol stage. The completed study should report reasons for non-participation or exclusion when available. |
| Results | 13c | Consider use of a flow diagram | Not applicable at protocol stage | Not applicable at protocol stage. A participant flow diagram is recommended for the completed study and could also be included as a planned recruitment flow diagram if desired. |
| Results | 14a | Give characteristics of study participants and information on exposures and potential confounders | Not applicable at protocol stage; planned in Statistical analysis | Not applicable at protocol stage. The protocol states that demographic characteristics and psychometric measures will be summarised descriptively and compared between groups. |
| Results | 14b | Indicate number of participants with missing data for each variable of interest | Not applicable at protocol stage; planned in Statistical analysis | Not applicable at protocol stage. |
| Results | 15 | Report numbers of outcome events or summary measures | Not applicable at protocol stage | Not applicable at protocol stage. The completed study should report NSSI and suicidality summary measures, and relevant scale distributions, separately for groups where applicable. |
| Results | 16a | Give unadjusted estimates and, if applicable, confounder-adjusted estimates and their precision. Make clear which confounders were adjusted for and why they were included | Not applicable at protocol stage; planned in Statistical analysis | Not applicable at protocol stage. The protocol plans unadjusted comparisons, SEM standardised coefficients, 95% confidence intervals, and p values. Actual estimates will be reported after data collection. |
| Results | 16b | Report category boundaries when continuous variables were categorized | Not applicable unless categorisation is used | Not applicable at protocol stage. Continuous variables are planned to be analysed as scale scores. If any variables are later categorised, the completed report should state cut points and rationale. |
| Results | 16c | If relevant, consider translating estimates of relative risk into absolute risk for a meaningful time period | Not applicable | Not applicable to the current protocol. |
| Results | 17 | Report other analyses done—eg analyses of subgroups and interactions, and sensitivity analyses | Not applicable at protocol stage; planned in Statistical analysis | Protocol-stage adaptation. Exploratory network analysis is prespecified as an additional analysis. Subgroup, interaction, and sensitivity analyses are not currently planned. |
| Discussion | 18 | Summarise key results with reference to study objectives | Abstract—Discussion; Discussion | Protocol-stage adaptation. Actual results are not available. The manuscript describes expected findings and relates them to the study objectives. |
| Discussion | 19 | Discuss limitations of the study, taking into account sources of potential bias or imprecision. Discuss both direction and magnitude of any potential bias | Discussion—limitations paragraph | Reported. Limitations include single-centre clinical recruitment, help-seeking sample, different recruitment contexts for controls, wide age range, and self-report bias. |
| Discussion | 20 | Give a cautious overall interpretation of results considering objectives, limitations, multiplicity of analyses, results from similar studies, and other relevant evidence | Discussion | Protocol-stage adaptation. The manuscript gives a cautious expected interpretation and connects the planned study to prior evidence and prevention implications. The completed report should interpret empirical results with attention to multiplicity and cross-sectional causal limitations. |
| Discussion | 21 | Discuss the generalisability (external validity) of the study results | Discussion—limitations paragraph | Reported. The manuscript notes that findings may be more representative of help-seeking or clinically referred young people than of individuals in the broader community, and that the clinical and control groups are recruited from different contexts. |
| Other information | 22 | Give the source of funding and the role of the funders for the present study and, if applicable, for the original study on which the present article is based | Declaration—Funding | Reported. Funding sources and the funders’ lack of role in study design, data collection, analysis, interpretation, writing, and submission are stated. |
